# A Model-Informed Drug Development (MIDD) Approach for a Low Dose of Empagliflozin in Patients with Type 1 Diabetes

**DOI:** 10.3390/pharmaceutics13040485

**Published:** 2021-04-02

**Authors:** Curtis K. Johnston, Rena J. Eudy-Byrne, Ahmed Elmokadem, Valerie Nock, Jan Marquard, Nima Soleymanlou, Matthew M. Riggs, Karl-Heinz Liesenfeld

**Affiliations:** 1 Metrum Research Group, Tariffville, CT 06081, USA; curtisj@metrumrg.com (C.K.J.); renae@metrumrg.com (R.J.E.-B.); ahmede@metrumrg.com (A.E.); mattr@metrumrg.com (M.M.R.); 2 Boehringer Ingelheim International GmbH, 55216 Ingelheim, Germany; valerie.nock@boehringer-ingelheim.com; 3 Boehringer Ingelheim Pharmaceuticals Inc., Ridgefield, CT 06877, USA; jan.marquard@boehringer-ingelheim.com (J.M.); nima.soleymanlou@boehringer-ingelheim.com (N.S.)

**Keywords:** Bayesian modeling, clinical trial simulations, SGLT2 inhibitor, supportive evidence, type 1 diabetes

## Abstract

In clinical trials, sodium-glucose co-transporter (SGLT) inhibitor use as adjunct to insulin therapy in type 1 diabetes (T1D) provides glucometabolic benefits while diabetic ketoacidosis risk is increased. The SGLT2 inhibitor empagliflozin was evaluated in two phase III trials: EASE-2 and EASE-3. A low, 2.5-mg dose was included in EASE-3 only. As the efficacy of higher empagliflozin doses (i.e., 10 and 25 mg) in T1D has been established in EASE-2 and EASE-3, a modeling and simulation approach was used to generate additional supportive evidence on efficacy for the 2.5-mg dose. We present the methodology behind the development and validation of two modeling and simulation frameworks: M-EASE-1, a semi-mechanistic model integrating information on insulin, glucose, and glycated hemoglobin; and M-EASE-2, a descriptive model informed by prior information. Both models were developed independently of data from EASE-3. Simulations based on these models assessed efficacy in untested clinical trial scenarios. In this manner, the models provide supportive evidence for efficacy of low-dose empagliflozin 2.5 mg in patients with T1D, illustrating how pharmacometric analyses can support efficacy assessments in the context of limited data.

## 1. Introduction

Type 1 diabetes (T1D) is an autoimmune disorder causing serious metabolic dysregulation characterized by insulin deficiency and substantial reduction in life expectancy [1,2]. Inhibitors of sodium-glucose co-transporters 1 (SGLT1) and 2 (SGLT2) have been shown to improve glycemic control and result in weight loss in patients with T1D [3,4,5,6,7]. Although these agents have been shown to improve glucometabolic outcomes in the context of clinical trials, their use is associated with an increased risk of diabetic ketoacidosis [6,7,8]. As a consequence, while dapagliflozin and sotagliflozin have been approved for the treatment of T1D in Europe, no SGLT2 inhibitors have been approved for this indication in the United States, owing to the increased risk of diabetic ketoacidosis.

Empagliflozin is a highly selective SGLT2 inhibitor that has been approved for use in adults with type 2 diabetes (T2D). The benefit–risk profile of empagliflozin in T1D was evaluated in the Empagliflozin as Adjunctive to Insulin Therapy (EASE) clinical program, which included two international, multicenter, phase III, randomized, double-blind, placebo-controlled, parallel-group sister trials of once-daily oral empagliflozin doses conducted over 52 weeks (EASE-2) and 26 weeks (EASE-3) [8,9,10]. In phase III trials, empagliflozin significantly improved glycemic control and resulted in significant reductions in weight, daily insulin dose, and blood pressure; severe hypoglycemic episodes were rare and occurred with similar frequency with empagliflozin and placebo. The EASE-2 trial included empagliflozin at doses of 10 and 25 mg, while the EASE-3 trial also included a unique, lower, 2.5-mg empagliflozin dose; this 2.5-mg dose resulted in improvements of glucometabolic endpoints. The 4-week phase II trials (EASE-1 [8] and EASE-J [9]) also assessed the efficacy of the empagliflozin 2.5-mg dose, alongside the 10- and 25-mg empagliflozin doses. In these trials, reductions in plasma glucose were observed with all empagliflozin doses [10].

Typically, two separate confirmatory and well-controlled clinical trials are needed to gain regulatory approval for a sought indication. However, the US Food and Drug Administration (FDA) Modernization Act of 1997 allows determination of substantial evidence of effectiveness to be based on “data from one adequate and well-controlled investigation and confirmatory evidence” [11]. In some cases, this confirmatory evidence can be provided by other types of evidence, including exposure–response studies [12,13]. Exposure–response analyses done by population pharmacokinetic/pharmacodynamic (PopPK/PD) modeling may be used to generate primary evidence of a drug’s efficacy. It has been suggested that these data could be used as supportive, or even pivotal, evidence of effectiveness [14]. 

The objective of this work was to generate additional supportive evidence on efficacy and to investigate factors that drive the variability in treatment effect on glycated hemoglobin (HbA1c). This was done using a descriptive exposure–response model to simulate the longitudinal placebo-adjusted HbA1c change from baseline in EASE-2 up to 52 weeks, and to assess the impact of covariates on the exposure–response relationship for HbA1c (M-EASE-2). To gain further confidence in the predictions, confirm the simulation results, and investigate the impact of insulin titration, a second, independent modeling approach was implemented. This approach, referred to herein as M-EASE-1, was a semi-mechanistic, exposure–response model used to simulate the longitudinal placebo-adjusted HbA1c change from baseline in EASE-2 up to 52 weeks and to assess the impact of adjusted vs. fixed insulin dose treatment on HbA1c. 

The analyses presented here focus on the efficacy of empagliflozin. Modeling of the important safety endpoint, diabetic ketoacidosis (DKA), was not possible, as although information on increases in beta-hydroxybutyrate (BHB) was available, information on precipitating factors, such as non-adherence, insulin restriction/omission, disordered eating behaviors or infections, was not available in patients who did not experience such an event. The clinical effects of empagliflozin on HbA1c and BHB levels as determined by modeling efforts have been discussed and put into clinical perspective previously [15]. Here, we present the detailed methodology for the development, validation and clinical trial simulations behind the two modeling and simulation frameworks: M-EASE-1, a semi-mechanistic model integrating information on insulin, glucose, and glycated hemoglobin; and M-EASE-2, a descriptive model informed by prior information. 

## 2. Materials and Methods

### 2.1. Included Study Data

Both modeling frameworks were informed by data from EASE-1 (a 4-week phase II study that included empagliflozin 2.5, 10, and 25 mg once-daily (qd) treatment arms) and EASE-2 (a 52-week study that included empagliflozin 10 and 25 mg qd treatment arms) [8,16], while data from EASE-3, which included the low 2.5-mg dose of empagliflozin, was only used for evaluation of the model (Table 1). This was done to generate evidence of efficacy independent of the effect observed in EASE-3. A detailed description of the phase III study methodology, including the insulin intensification protocol used, has been previously published [8].

### 2.2. Software

The PopPK and PopPK/PD analyses were conducted via nonlinear mixed effects modeling using the nonlinear mixed effects modeling (NONMEM^®^) software versions 7.3 and 7.4 (ICON Development Solutions, Hanover, MD, United States), respectively. The PK and semi-mechanistic exposure–response models employed first-order conditional estimation with *ƞ–Є* interaction. The descriptive exposure–response model utilized a Markov chain Monte Carlo (MCMC) Bayesian estimation; specifically, a Hamiltonian Monte Carlo estimation with the No-U-Turn sampler was utilized with default settings. All data processing and graphical generation were conducted using R version 3.3 (www.r-project.org, The R Foundation, Vienna, Austria). Simulations were performed using the mrgsolve package (version 0.9.0., Metrum Research Group, Tariffville, CT, USA) [17] in R.

### 2.3. Population PK Model

#### 2.3.1. Model Development

The PopPK model used in this analysis was adapted from a previous PopPK modeling study [18] and updated using plasma empagliflozin concentrations, dosing history, observation times, and covariate factors from empagliflozin patients in EASE-1 and EASE-3 for initial model development, with EASE-2 data used as an external evaluation dataset. The best-performing model was re-estimated using combined data from all studies to predict individual patient exposures (area under the curve at steady-state; *AUC_ss_*) for the exposure–response analyses.

The analysis used a full covariate modeling approach [19] that emphasized parameter estimation. Covariates incorporated into the full PopPK model included age, weight, sex, total protein, alkaline phosphatase, smoking status, total daily insulin dose (TDID), and estimated glomerular filtration rate (eGFR).

#### 2.3.2. Model Evaluation

Internal and external evaluations of the model were performed using posterior predictive checks (PPCs) via 500 Monte Carlo simulation replicates. The results were summarized as longitudinal visual predictive checks (VPCs) [20] and landmark checks of C_max_ and C_min_ for individual patients at steady-state. In addition, the precision of parameters was estimated via a nonparametric bootstrap (250 iterations) [21,22].

### 2.4. Exposure–Response Analyses

#### 2.4.1. Model Development: Semi-Mechanistic Model (M-EASE-1)

The time course of HbA1c was initially planned to be estimated via an indirect-response model but, due to identifiability issues, a simpler direct-effect model was implemented. This was driven by the information content of the data (i.e., the timing of the endpoint collections did not support identification of indirect-response model parameters). The direct-effect model assumed that the exposure-related effect of empagliflozin was at steady-state (and so was time-independent) prior to the collection of the first HbA1c sample collections at week 4 of the studies. 

**Figure 1:**
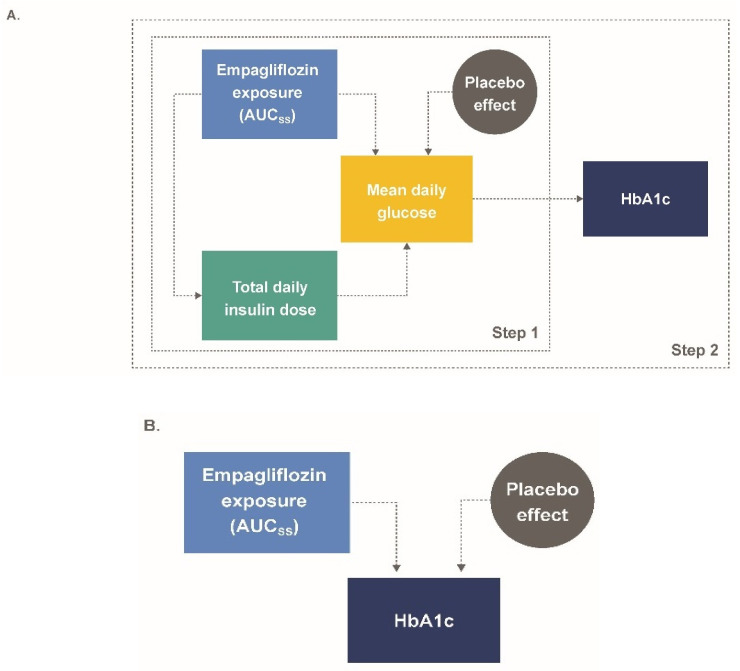
(**A**) M-EASE-1 and (**B**) M-EASE-2 schematic final model structure. *AUC_ss_*, area under the curve at steady-state; HbA1c, glycated hemoglobin; MDG, mean daily glucose; TDID, total daily insulin dose.

Exposure–response relationships between longitudinal HbA1c, TDID, and mean daily glucose (MDG) measurements as functions of empagliflozin *AUC_ss_* were parametrically modeled in a stepwise fashion (Figure 1A). First, changes in insulin (expressed as TDID) were described for the single endpoint TDID as a function of empagliflozin exposure. Second, to assess the impact of changes in TDID on glucose levels, a model was developed for the two endpoints (TDID, MDG) based on placebo data. Parameters for this model were fixed, and the previously estimated effect of empagliflozin exposure on TDID was included, which in turn resulted in changes in MDG. In addition, a placebo effect was added to drive changes in MDG. Based on this model, individual MDG profiles were derived and parameters affecting the time course of HbA1c were estimated. The relationships of the final insulin-MDG and HbA1c model equations were:

(1) $$TDID_{i,j}=TDID_{t0,i}\cdot Inc_{i}\cdot \left(1-\frac{E_{max,TDID,i}\cdot AUC_{ss,i}}{AUC_{50,TDID}+AUC_{ss,i}}\right)$$

(2) $$MDG_{i,j}=MDG_{t0,i}\cdot {\left(\frac{TDID_{i,j}}{TDID_{t0,i}}\right)}^{\theta_{a}}+PBO_{MDG}\cdot TIME-\left(\frac{E_{max,MDG,i}\cdot AUC_{ss,i}}{AUC_{50,MDG}+AUC_{ss,i}}\right)$$

(3) $$HbA1c_{i,j}=HbA1c_{t0,i}\cdot {\left(\frac{MDG_{i,j}}{MDG_{t0,i}}\right)}^{\theta_{b}\cdot exp^{\eta_{2}}}$$

In these equations, *TDID_i_*_,*j*_ represents the predicted TDID for a given patient at a given time; *TDID_t_*_0,*i*_ represents predicted baseline insulin dose for a given patient (*i*); *Inc_i_* represents a scale parameter reflecting the amplitude for insulin dose adjustment (applies only to EASE-1 during the first week of treatment); *E_max,TDID_*_,*i*_ represents the maximal effect parameter for empagliflozin *AUC_ss_* on TDID achieved; *AUC*_50,*TDID*_ represents the *AUC_ss_* at which half the maximal effect of empagliflozin on TDID is achieved; *AUC_ss,i_* represents the individual empirical Bayes estimates of *AUC_ss_*; *PBO_MDG_* is the time-dependent MDG placebo effect; *E_max,MDG_*_,*i*_ represents the maximal effect parameter for empagliflozin *AUC_ss_* on MDG achieved; *AUC*_50,*MDG*_ represents the *AUC_ss_* at which half the maximal effect of empagliflozin on MDG is achieved; *HbA1c_t_*_0,*i*_ represents the predicted baseline HbA1c for a given patient; and *MDG_i_*_,*j*_ represents the MDG, calculated from *AUC*_0–24,*glu*,*i*,*j*/24_ for a given subject and time. 

#### 2.4.2. Model Evaluation: Semi-Mechanistic Model (M-EASE-1)

Internal and external model evaluation consisted of PPCs summarized as longitudinal VPCs for the time courses of TDID, MDG, and HbA1c, and landmark checks at each observation timepoint for HbA1c (Table 2).

#### 2.4.3. Applied Simulations: Semi-Mechanistic Model (M-EASE-1) 

To assess the effect of insulin adjustment, simulations with and without an empagliflozin exposure effect on TDID (hypothetical stable insulin) were conducted. Clinical practice would not allow for stable insulin without an increased risk of hypoglycemia. This simulation can show the net effect in T1D patients. In addition, simulations of the placebo-adjusted HbA1c response for a 2.5 mg dose in the EASE-2 population were conducted to generate additional evidence of efficacy, independent of EASE-3 (Table 2).

#### 2.4.4. Model Development: Descriptive Model (M-EASE-2)

As with M-EASE-1, initial considerations for a more complex indirect-response model were exchanged instead with direct exposure–response models. As EASE-2 and EASE-1 mainly provided information from the 10- and 25-mg doses, an informative prior for *AUC*_50_ (*AUC_ss_* leading to 50% of maximal effect) was used from a previously conducted analysis in patients with T2D (*AUC*_50_ of 704 nmol·h/L) [23]. For all other parameters, noninformative prior distributions were utilized, with normal distributions used for the fixed-effect parameters and inverse Wishart distributions (degrees of freedom (df) = no. diagonal elements of OMEGA) for the random effects. For each model, four separate chains were run with the initial estimates varied for all parameters. Each chain was run with a total of 2000 warm-up samples followed by 10,000 post-warm-up samples. The observed placebo effect was described by a linear function of time:

(4) $$Baseline_{HbA1c,i}=\theta_{a}\cdot \overset{m}{\underset{1}{\prod }}{\left(\frac{cov_{mi}}{ref_{m}}\right)}^{\theta_{\left(m+a\right)}}\cdot \overset{p}{\underset{1}{\prod }}\theta_{\left(p+m+a\right)}^{cov_{pi}}\cdot exp^{\eta_{z}}$$

(5) $$AUC_{50}=\theta_{b}$$

(6) $$E_{max,i}=\theta_{c}\cdot \overset{m}{\underset{1}{\prod }}{\left(\frac{cov_{mi}}{ref_{m}}\right)}^{\theta_{\left(m+c\right)}}\cdot \overset{p}{\underset{1}{\prod }}\theta_{\left(p+m+c\right)}^{cov_{pi}}\cdot exp^{\eta_{s}}$$

(7) $$Placebo=\theta_{d}$$

(8) $$HbA1c_{i,j}=Baseline_{HbA1c,i}-\left(\frac{E_{max,i}\cdot AUC_{ss,i}}{AUC_{50}+AUC_{ss,i}}\right)+Placebo\cdot TIME$$

In these equations, *Baseline_HbA1c,i_* is the patient-specific predicted HbA1c level at baseline; *AUC*_50_ is the AUC at which half the maximal effect of empagliflozin on HbA1c is seen; *cov_mi_* is the individual covariate value for the continuous covariate “*m*”; *cov_pi_* is the individual covariate value for the categorical covariate “*p*”; *ref_m_* is the population covariate value for the continuous covariate “*m*”; *η*: are individual-specific inter-subject random effects; *E_max,i_* is the patient-specific predicted maximal effect parameter on HbA1c; *Placebo* is an additive placebo effect as a function of time; and *AUC_ss,i_* is the individual empirical Bayes estimate of empagliflozin exposure *AUC_ss_*. 

Using the final model (Figure 1B), a full covariate analysis was conducted; preselected covariates included for baseline HbA1c were baseline weight, gender, baseline TDID, multiple daily injections (MDI) vs. continuous subcutaneous insulin infusion (CSII), and baseline eGFR; *E_max_* covariates were baseline weight, gender, baseline HbA1c, baseline TDID, MDI vs. CSII, and baseline eGFR; placebo covariates were gender and MDI vs. CSII. The key assumptions of the final model were considered and tested (Supplementary Materials Table S1).

#### 2.4.5. Model Evaluation: Descriptive Model (M-EASE-2) 

To assess the convergence of the MCMC chains, trace plots of the posterior samples, posterior histograms, the calculated effective sample sizes, and the Gelman–Rubin convergence diagnostic were evaluated [24]. Internal and external model evaluation consisted of PPCs summarized as longitudinal VPCs of the HbA1c response and landmark checks at each observation timepoint (see Table 2 for details).

#### 2.4.6. Applied Simulations: Descriptive Model (M-EASE-2) 

Simulations were conducted to assess the impact of covariates on HbA1c lowering. A specific emphasis on change in HbA1c as a function of baseline HbA1c was evaluated. 

To generate additional evidence of efficacy for empagliflozin 2.5 mg qd administered to patients with T1D, independent from the EASE-3 clinical results, simulations of the placebo-adjusted HbA1c response for a 2.5-mg dose in the EASE-2 population were conducted. To assess the impact of the prior information on the model predictions, a sensitivity analysis was performed [24]. Specifically, varying levels of informativeness (magnitude of variance for prior distribution: fixed to zero, inflated by 10-, 50-, and 100-fold, and an extreme noninformative prior) and alternate location parameters for the prior distribution (extreme large value (22,026 nmol·h/L), extreme small value (0.00005 nmol·h/L), and a 50% increase and decrease were utilized for the final model to assess how dependent the model predictions were on the choice of prior (Table 2).

## 3. Results

### 3.1. Population PK Model 

The study population included 1241 patients (614 males, 627 females) from EASE-1, -2, and -3 on active treatment. A two-compartment model with first-order elimination and a sequential zero- and first-order oral absorption and a lag time described the base model for the PopPK of empagliflozin (Table S2). Structural parameters were comparable to previous modeling conducted in T2D [23]; no covariates were found to have clinically relevant impacts on exposure (Figure S1). Evaluation of the PopPK model by PPCs and a nonparametric bootstrap demonstrated that the model provided an adequate description of the data; re-estimation with the inclusion of EASE-2 data produced similar results. 

### 3.2. Semi-Mechanistic Model (M-EASE-1)

The model included 796 patients (534 empagliflozin patients and 262 placebo patients), with a total of 4824 HbA1c observations, 189,182 TDID observations, and 4243 MDG observations. Overall, TDID was well described using a proportional *E_max_* function driven by *AUC_ss_* (Equation (1)). MDG was affected by three components: empagliflozin exposure expressed as a direct-response *E_max_* function (Equation (2)); a linear time-dependent placebo effect (Equation (3)); and TDID profiles derived from the first part of the model development. Changes in HbA1c were driven by changes in MDG as predicted in the second step (Figure 1A). In general, the population parameters, inter-individual, proportional and additive residual variability estimates were precisely estimated and were representative of the observed data (Table 3).

With the exception of the baseline HbA1c effect on *E_max,MDG_* and eGFR effects on baseline HbA1c, which were excluded due to model instability, all pre-specified covariate effects were maintained in the final version of the models. However, all estimated covariate effects were either negligible in magnitude or estimated with poor precision (Table S3). 

### 3.3. Model Evaluation: Semi-Mechanistic Model (M-EASE-1) 

The developed models were able to accurately capture the time courses of TDID, MDG, and HbA1c across each treatment arm for the EASE-1 and EASE-2 data. Similarly, the external evaluation using out-of-sample predictions from EASE-3 indicated that the model adequately captured the time courses of TDID, MDG, and HbA1c across all dose groups including the 2.5-mg treatment arm (Figure S2).

### 3.4. Applied Simulations: Semi-Mechanistic Model (M-EASE-1) 

**Figure 2:**
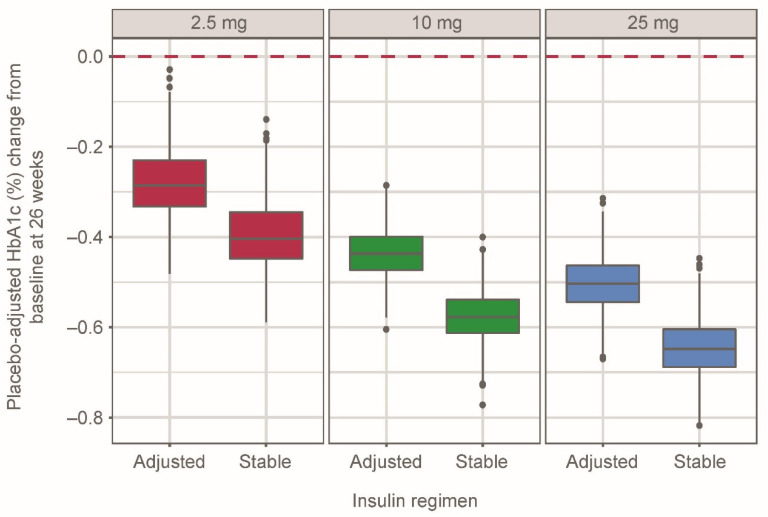
Placebo-adjusted simulated HbA1c (%) change from baseline at week 26 in 500 patients per empagliflozin dose group for each regimen, grouped by empagliflozin dose and insulin regimen using the semi-mechanistic model. Distributions represent simulated median values from 500 replicates. Whiskers represent 1.5× interquartile range. Black dots indicate simulated data outside of 1.5× interquartile range.

The simulated median (95% CI) placebo-adjusted HbA1c change from baseline at week 26 for empagliflozin 2.5 mg qd was −0.29% (−0.40%, −0.10%) and −0.40% (−0.53%, −0.23%) with adjusted and stable insulin therapy, respectively (Figure 2). In the 10- and 25-mg groups, median (95% CI) placebo-adjusted changes in HbA1c at week 26 were −0.44% (−0.55%, −0.33%) and −0.50% (−0.63%, −0.38%), respectively, with adjusted insulin therapy and −0.58% (−0.69%, −0.46%) and −0.65% (−0.78%, −0.52%), respectively, with stable insulin therapy.

**Figure 3:**
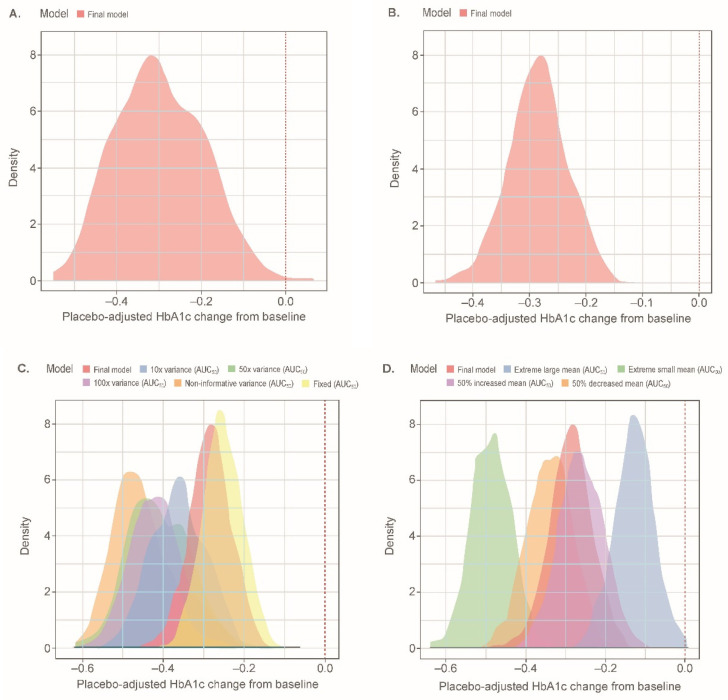
Distributions of simulated median placebo-adjusted HbA1c (%) change from baseline at week 26 for a 2.5-mg empagliflozin treatment arm in the EASE-2 population: (**A**) with the semi-mechanistic model; (**B**) with the descriptive model; (**C**) with different prior variances; (**D**) with different prior means. Distributions represent simulated median values for 500 replicates including parameter uncertainty for each respective model. Extreme large mean: 22,026 nmol·h/L; Extreme small mean: 0.00005 nmol·h/L. *AUC*_50_, *AUC_ss_* leading to 50% of maximal effect; *AUC_ss_*, area under the curve at steady-state; HbA1c, glycated hemoglobin.

Simulations of a 2.5-mg dose in the EASE-2 population resulted in a median (95% CI) placebo-adjusted HbA1c change from baseline of −0.31% (−0.48%, −0.10%) at week 26; the effect was predicted to be sustained over 52 weeks. The distribution of median values at week 26 across 500 study replicates was relatively small; 81.2% of study replicates showed an HbA1c change of at least −0.20% and 67.0% of the study replicates showed an HbA1c change of at least −0.25% (Figure 3A). 

### 3.5. Descriptive Model (M-EASE-2)

The M-EASE-2 descriptive model included data from 391 males and 405 females (total 796; 534 on empagliflozin, 262 on placebo), with a total of 4899 observations of HbA1c (4750 in EASE-2 and 149 in EASE-1). There was relative balance between categorical covariates, with the majority of patients using MDI (64%). The 95th percentile intervals for age, baseline weight, HbA1c, eGFR, and baseline TDID (IDB) were 21–69 years, 55–125 kg, 7.2–9.5%, 57–127 mL/min/1.73 m^2^, and 0.370–1.30 IU/kg, respectively.

There were no apparent correlations between continuous covariates with the exception of age and eGFR, and no overt trends in continuous covariates were observed except for a slight trend towards higher baseline HbA1c with increased IDB. A simple *E_max_* model driven by *AUC_ss_* with a linear placebo effect as a function of time was utilized (Figure S3). A proportional *E_max_* model was also tested but did not perform as well as the nonproportional model. The estimated maximal decrease from baseline as a function of empagliflozin exposure was 0.58%, and the *AUC_50_* was estimated to be 498 nmol·h/L (Table 3). Patients receiving CSII had larger placebo increases in HbA1c (47% greater) relative to patients receiving MDI. In addition, greater baseline HbA1c and eGFR values were estimated to increase *E_max_* (e.g., a 32% increase when baseline HbA1c ranged from 7.2% to 9.5%; a 50% increase when eGFR ranged from 54 to 120 mL/min/1.73 m^2^).

### 3.6. Model Evaluation: Descriptive Model (M-EASE-2) 

The majority of parameters were sampled with limited autocorrelation as demonstrated by the trace plots, Rhat values of approximately 1.0, and effective sample sizes all at approximately 1000 or higher. The developed model was able to accurately capture the time course of HbA1c across each treatment arm for the data used to initially develop the model. Similarly, when predicting into the external data across all the timepoints at the median value, no consistent trend of over- or underpredicting was observed (Figure S4). 

### 3.7. Applied Simulations: Descriptive Model (M-EASE-2) 

Simulations to illustrate the impact of baseline HbA1c on change in HbA1c were performed; for a 2.5-mg qd dose, a median placebo-adjusted change in HbA1c at 26 weeks relative to baseline of −0.28% vs. −0.32% was predicted for a baseline HbA1c of 8.0% and 9.0%, respectively (Table S4 and Figure S5).

Simulations of a 2.5-mg dose in the EASE-2 population resulted in a median (95% CI) placebo-adjusted HbA1c change from baseline of −0.29% (−0.39%, −0.19%) at week 26; the effect was predicted to be sustained over 52 weeks. The distribution of median values at week 26 across 500 study replicates was relatively narrow; 95.0% of study replicates showed an HbA1c change of at least −0.20%, and 76.8% of the study replicates showed an HbA1c change of at least −0.25% (Figure 3B). Simulated population exposures for the 2.5-mg dose were centered at the estimated *AUC*_50_ (Figure S6). 

The simulated distribution of placebo-adjusted HbA1c change from baseline for the final model at a dose of 2.5 mg falls close to the distribution generated when fixing the *AUC*_50_ value to the estimated value from patients with T2D (Figure 3C). This was in contrast to the distributions obtained when increasing the variance on the parameter. Increasing the variance resulted in greater changes from baseline, with a noninformative prior resulting in median values of −0.46% (Table S5) and a smaller estimated *AUC*_50_ value. As expected, increasing the mean of the prior distribution resulted in decreased drug effects and the converse resulted in increased drug effects (Figure 3D). 

## 4. Discussion

Two independent approaches were followed in order to address different questions and to gain confidence in the clinical trial simulations that were conducted to provide supportive evidence. On the one hand, the impact of insulin adjustment should be quantified in order to compare the HbA1c reduction to patients with T2D. This was only possible with the semi-mechanistic model. On the other hand, factors that drive the variability in treatment should be quantified. This could only be done with the descriptive model. To provide supportive evidence for the efficacy of the 2.5-mg dose of empagliflozin in patients with T1D, independent of the EASE-3 clinical trial, both PopPK/PD models were developed using data from EASE-1 and EASE-2 only. To assess the utility of the developed models to reliably predict the long-term effect of the 2.5-mg dose, EASE-3 was used as an external evaluation dataset. As predictions into independent data provide a stringent test of a given model’s assumptions and predictive ability, the authors considered this approach to offer a more rigorous evaluation relative to pooling all available data to estimate a model and then using it to simulate supporting efficacy data. However, there were limitations with the descriptive model as the majority of data used for model building was at the 10- and 25-mg dose levels, both of which were expected to be near the plateau of the exposure–response relationship. To address the issue, estimation was conducted in a Bayesian framework, borrowing information from a prior analysis of fasting plasma glucose (FPG) and HbA1c lowering in patients with T2D (drug effect included on degradation of FPG). 

Using an informative Bayesian prior necessitated the execution of a sensitivity analysis to assess the impact of the employed prior on the model’s predictive performance. The selected variance for the final model trended to the conservative side of the predicted placebo-adjusted HbA1c change from baseline at 52 weeks, as increases in variance predicted larger drug effects. This occurred due to the estimated *AUC*_50_ being lower with increased variance for the prior. When adjusting the mean of the prior distribution, increases resulted in decreased drug effects and decreases resulted in increased drug effects. The results of the sensitivity analysis demonstrate that the prior that was utilized for the *AUC*_50_ parameter provided the requisite support for estimation of the *AUC*_50_, and resulted in conservative estimates of the 2.5 mg empagliflozin effect on HbA1c that were consistent with EASE-3. 

As part of a regulatory review, an additional sensitivity analysis was conducted for the descriptive modeling whereby the model was estimated without the use of a Bayesian prior [25]. Using this model, the median simulated placebo-adjusted change from baseline HbA1c for a typical patient in EASE-2 receiving a 2.5-mg dose was −0.43% (95% CI −0.52%, −0.31%) at 26 weeks. This value is approximately 50% larger than what was observed in EASE-3 (−0.28%) [8] but was consistent with the predictions observed during the sensitivity analysis when using a noninformative prior, as Bayesian estimation can approximate maximum likelihood estimates under conditions of noninformative priors [24]. As an alternative, a conservative approach considering the lower bound of the 95% CI of the model prediction as a “worst-case-scenario” was assessed. In this case, the median value was simulated to be −0.23% (95% CI −0.38% to −0.05%), which was similar to the empirical model’s prediction and the observed effect in EASE-3 [25]. Even though the employment of an informative Bayesian prior is not standard to support evidence of efficacy, the FDA concluded during an Endocrinologic and Metabolic Drugs Advisory Committee meeting, “we believe this model provides supportive evidence of the effect size for the 2.5-milligram dose independent of EASE-3” [25].

The semi-mechanistic model allowed for the investigation of the interplay between empagliflozin exposures, changes in insulin dose and MDG, and their joint effect on HbA1c lowering. Such a framework permitted the evaluation of insulin titration on changes in HbA1c time course. As expected, a greater magnitude of percentage change from baseline HbA1c resulted from a stable insulin regimen (30% greater decrease), relative to an adjusted insulin regimen. Despite relying upon an alternate set of assumptions and level of complexity, the semi-mechanistic model provided comparable predictions of efficacy for a 2.5-mg dose of empagliflozin relative to the descriptive model and the effect observed in EASE-3.

Some limitations are noted with these analyses. First, the descriptive model relied upon an informative Bayesian prior from a different disease population and from a different endpoint. The decision to use a prior was based upon exposure–response modeling performed for urinary glucose excretion (UGE), a short-term marker for efficacy of SGLT2 inhibitors, in patients with T1D and T2D [18]. The analysis showed that the estimated PD parameters for patients with T1D and T2D were comparable with only slight differences overall. Those differences lead to an increase in UGE in patients with T1D relative to patients with T2D at similar exposures but a comparable shape of the exposure–response relationship overall. As the exposure–response for UGE, a marker for glucose lowering, was comparable between the two populations, we assumed that information on *AUC*_50_ from an exposure–response analysis for FPG and HbA1c conducted in patients with T2D could be used as informative prior. Despite these assumptions, the insulin dose reductions that occurred while on treatment could affect the UGE in patients with T1D differently from T2D. Accordingly, we conducted an external evaluation to assess the adequacy of the model predictions and a sensitivity analysis (described above) to understand the operating characteristics of the model predictions as a function of the prior. The external evaluation demonstrated the ability of the estimated model to independently predict HbA1c outcomes across all dose groups and timepoints in EASE-3, and the sensitivity analysis highlighted the necessity for the prior as predictions from a model estimated without the prior information resulted in highly biased predictions of HbA1c changes. 

A second limitation of these analyses was the inability to use indirect response models to characterize the time courses of the PD endpoints. For both models, this was due to issues with identifiability, as the nadir of drug effect occurred at the first on-treatment observation for HbA1c. Reaching the nadir of the drug effect within 4 weeks of the start of therapy had not been expected based on earlier calculated half-lives for HbA1c [23]. The most likely reason was the impact of insulin intensification during the run-in period of the phase III trials. This might also explain the drop in MDG and HbA1c seen in the placebo group after four weeks. 

Third, the use of a time-dependent linear placebo response to describe changes in HbA1c in both models has no physiological basis. This linear placebo model slightly overpredicts the observed HbA1c values at four weeks (Figures S2A and S4) in the placebo group and is not suitable to reliably extrapolate absolute HbA1c values beyond 52 weeks. Alternative implementations included asymptotic functions of time with and without lagged onsets. However, none of these tested models were stably estimated or resulted in improved numerical performance. Despite the simplicity and empirical implementation, the linear placebo models adequately predicted observed increases in HbA1c over time (except for 4 weeks) and across dose groups. The adequate prediction of observed data in EASE-3 at 26 weeks (model qualification) allows answering of the key objective, the reliable prediction of placebo-adjusted changes in HbA1c in EASE-2 at 26 and 52 weeks. 

Lastly, in the context of an overall efficacy and safety assessment, this evaluation does not inform on safety aspects. Although quantitative BHB levels as precursor for DKA and marker for safety could be modeled and showed a clear exposure–response relation, DKA events as clinical relevant outcome could not be modeled because of low event numbers and an inability to model relevant precipitating factors (such as infections or pump malfunction) [8].

## 5. Conclusions

This work provides detailed methodology (model development, validation and clinical trial simulations) for two different pharmacometric modeling approaches that independently confirm the observed efficacy for a low dose of empagliflozin 2.5 mg in patients with T1D, which was observed in EASE-3. Using the models to simulate the outcome of a 2.5-mg dose in the EASE-2 study population provided supportive evidence of the efficacy of a low dose of empagliflozin. As such, they may be considered to be examples of high-impact analyses as defined by Marshall et al. [26], illustrating how pharmacometric analyses can support efficacy assessments in the context of limited data. 

## Figures and Tables

**Table 1:** Overview of included studies.

Study	Phase	Description	Study Population	Patient Demographics: Median (95% Confidence Interval)
EASE-1 [16]	II	Once-daily EMPA 2.5, 10, 25 mg, or placebo for 28 days	75 patients with T1D	Age (y): 43.0 (21.7–61.0) Weight (kg): 79.0 (52.0–107) eGFR (mL/min/1.73 m^2^): 102 (78.7–128) TDID (IU/kg): 0.640 (0.361–1.13) HbA1c (%): 8.20 (7.18–9.61)
EASE-2 [8]	III	Once-daily EMPA 10, 25 mg, or placebo for 52 weeks	721 patients with T1D	Age (y): 46.0 (21.0–69.0) Weight (kg): 84.0 (55.0–126) eGFR (mL/min/1.73 m^2^): 97.0 (57.0–127) TDID (IU/kg): 0.680 (0.370–1.34) HbA1c (%): 8.00 (7.20–9.50)
EASE-3 [8] ^a^	III	Once-daily EMPA 2.5, 10, 25 mg, or placebo for 26 weeks	948 patients with T1D	Age (y): 43.0 (21.0–69.0) Weight (kg): 81.0 (55.0–121) eGFR (mL/min/1.73 m^2^): 99.0 (54.7–129) TDID (IU/kg): 0.660 (0.360–1.24) HbA1c (%): 8.10 (7.20–9.50)

^a^ Used for population pharmacokinetic/pharmacodynamic model evaluation only. eGFR, estimated glomerular filtration rate; EMPA, empagliflozin; HbA1c, glycated hemoglobin; T1D, type 1 diabetes; TDID, total daily insulin dose.

**Table 2:** Summary of simulation scenarios for model evaluation and simulation of M-EASE-1 and -2.

Scenario	Data Used	Description
M-EASE-2 descriptive and M-EASE-1 semi-mechanistic		
Internal PPC	EASE-1 and EASE-2	PPCs were generated via 500 Monte Carlo simulation replicates, which were generated and summarized as longitudinal VPCs and landmark checks at each observation timepoint. Parameter uncertainty was incorporated via the covariance matrix and the posterior distribution for M-EASE-1 and M-EASE-2, respectively
External “out-of-sample” PPC	EASE-3	
M-EASE-2 descriptive and M-EASE-1 semi-mechanistic		
Sensitivity analysis and predictions of response	EASE-2 (empagliflozin-treated patients)	Steady-state exposures at the 2.5-mg dose level were generated using individual-specific empirical Bayes estimate of the PK parameters. 239 patients from the M-EASE-2 population were randomly sampled, without replacement, for each of the 500 simulations. Parameter uncertainty was incorporated via the covariance matrix and the posterior distribution for M-EASE-1 and M-EASE-2, respectively
M-EASE-1 semi-mechanistic		
Stable vs. adjustable insulin	EASE-1, EASE-2, EASE-3	Each of the 500 simulations included 500 patients per dose group (placebo, EMPA 2.5, 10, and 25 mg qd) randomly sampled from the full data set (EASE-1, -2, and -3 populations); parameter uncertainty was incorporated via the covariance matrix. Two scenarios with and without an EMPA exposure effect on TDID (hypothetical stable insulin) were performed

EMPA, empagliflozin; PK, pharmacokinetic; PPC, posterior predictive check; qd, daily; TDID, total daily insulin dose; VPC, visual predictive check.

**Table 3:** Exposure–response parameters in M-EASE-1 and M-EASE-2.

**Key Parameters in M-EASE-2**	**Reference patient:** Male, MDI of insulin, baseline total daily dose = 0.660 U/kg, HbA1c = 8.1%, eGFR = 98 mL/min/1.73 m^2^, and baseline body weight = 82 kg.	
**Parameter**	**Estimate, median**	**95% CI**
Baseline HbA1c, %	8.14	8.07, 8.22
*AUC*_50_, nmol·h/L	498	296, 819
*E_max_*, %	0.579	0.491, 0.678
Placebo effect, %/h	2.61 × 10^−5^	1.96 × 10^−5^, 3.29 × 10^−5^
**Key parameters in M-EASE-1**	**Reference patient:** Male, eGFR = 99 mL/min/1.73 m^2^, body weight = 82 kg, cumulative MDG over 24 h, MDG = 4266 mg·day/dL	
**Parameter**	**Estimate, median**	**95% CI**
Baseline HbA1c, %	8.15	8.09, 8.21
*AUC*_50_ for TDID_EASE-1_, nmol·h/L	110	14.3, 836
*E_max_* for TDID	0.186	0.145, 0.238
*AUC*_50_ for MDG, nmol·h/L	370	83.9, 1630
*E_max_* for MDG, mg·day/dL	634	534, 753
WT_HbA1c_	−0.0258	−0.0528, 0.00125
SEX_HbA1c_	0.99	0.98, 1
γ_MDG EFF_	0.487	0.445, 0.532

*AUC*_50_, the *AUC_ss_* at which half the maximal effect of empagliflozin on TDID_EASE-1_ and MDG is achieved; *AUC_ss_*, area under the curve at steady-state; CI, confidence interval; EFF, power coefficient; eGFR, estimated glomerular filtration rate; *E_max_*, maximal effect parameter for empagliflozin *AUC_ss_* on TDID and MDG; HbA1c, glycated hemoglobin; MDG, mean daily glucose; MDI, multiple daily injections; SEX, patient gender; TDID, total daily insulin dose; WT, patient weight; γ, insulin effect.

## Data Availability

The sponsor of the study (Boehringer Ingelheim) is committed to responsible sharing of study reports, related documents, and study data. Researchers are invited to submit inquiries via the following website (https://trials.boehringer-ingelheim.com/).

## Supplementary Materials

The following are available online at https://www.mdpi.com/article/10.3390/pharmaceutics13040485/s1, Figure S1. Covariate forest plot on normalized *AUC_ss_* for the final PK model, Figure S2. M-EASE-1: External model evaluation for EASE-3 (out-of-sample) by longitudinal visual predictive check by dose for (A) HbA1c, (B) TDID, and (C) MDG, Figure S3. M-EASE-2: Placebo-adjusted simulated change in HbA1c at 26 weeks as a function of empagliflozin *AUC_ss_*_,_ Figure S4. M-EASE-2: Posterior predictive check for EASE-3 (out-of-sample) changes from baseline HbA1c by dose and week, Figure S5. M-EASE-2: Forest plot depicting the relative difference and precision of covariate effects on placebo-adjusted 26-week HbA1c change from baseline, Figure S6. Simulated empagliflozin *AUC_ss_* by dose using final population pharmacokinetic model, Table S1. Model assumptions, Table S2. Full covariate PK model: Summary of model parameter estimates, Table S3. M-EASE-1: Summary of final HbA1c/MDG/TDID model parameter estimates, Table S4. M-EASE-2: Full covariate model, summary of parameter estimates, Table S5: M-EASE-2: (A) Impact of prior variance on placebo-adjusted predicted median HbA1c change from baseline at 26 weeks; (B) Impact of prior mean on placebo-adjusted predicted median HbA1c change from baseline at 26 weeks; (C) M-EASE-2: Impact of prior variance on estimated *AUC*_50_ (nmol·h/L); and (D) M-EASE-2: Impact of prior mean on estimated *AUC*_50_ (nmol·h/L).

## References

[1] IDF Diabetes Atlas, 8th ed. https://www.idf.org/e-library/epidemiology-research/diabetes-atlas/134-idf-diabetes-atlas-8th-edition.html.

[2] Livingstone S.J., Levin D., Looker H.C., Lindsay R.S., Wild S.H., Joss N., Leese G., Leslie P., McCrimmon R.J., Metcalfe W. (2015). Estimated life expectancy in a Scottish cohort with type 1 diabetes, 2008–2010. JAMA.

[3] Dandona P., Mathieu C., Phillip M., Hansen L., Griffen S.C., Tschope D., Thoren F., Xu J., Langkilde A.M. (2017). Efficacy and safety of dapagliflozin in patients with inadequately controlled type 1 diabetes (DEPICT-1): 24 week results from a multicentre, double-blind, phase 3, randomised controlled trial. Lancet Diabetes Endocrinol..

[4] Dandona P., Mathieu C., Phillip M., Hansen L., Tschope D., Thoren F., Xu J., Langkilde A.M. (2018). Efficacy and safety of dapagliflozin in patients with inadequately controlled type 1 diabetes: The DEPICT-1 52-week study. Diabetes Care.

[5] Henry R.R., Thakkar P., Tong C., Polidori D., Alba M. (2015). Efficacy and safety of canagliflozin, a sodium-glucose cotransporter 2 inhibitor, as add-on to insulin in patients with type 1 diabetes. Diabetes Care.

[6] Mathieu C., Dandona P., Gillard P., Senior P., Hasslacher C., Araki E., Lind M., Bain S.C., Jabbour S., Arya N. (2018). Efficacy and safety of dapagliflozin in patients with inadequately controlled type 1 diabetes (the DEPICT-2 study): 24-week results from a randomized controlled trial. Diabetes Care.

[7] Sands A.T., Zambrowicz B.P., Rosenstock J., Lapuerta P., Bode B.W., Garg S.K., Buse J.B., Banks P., Heptulla R., Rendell M. (2015). Sotagliflozin, a dual SGLT1 and SGLT2 inhibitor, as adjunct therapy to insulin in type 1 diabetes. Diabetes Care.

[8] Rosenstock J., Marquard J., Laffel L.M., Neubacher D., Kaspers S., Cherney D.Z., Zinman B., Skyler J.S., George J., Soleymanlou N. (2018). Empagliflozin as adjunctive to insulin therapy in type 1 diabetes: The EASE trials. Diabetes Care.

[9] Shimada A., Hanafusa T., Yasui A., Lee G., Taneda Y., Sarashina A., Shiki K., George J., Soleymanlou N., Marquard J. (2018). Empagliflozin as adjunct to insulin in Japanese participants with type 1 diabetes: Results of a 4-week, double-blind, randomized, placebo-controlled phase 2 trial. Diabetes Obes. Metab..

[10] Famulla S., Pieber T.R., Eilbracht J., Neubacher D., Soleymanlou N., Woerle H.J., Broedl U.C., Kaspers S. (2017). Glucose exposure and variability with empagliflozin as adjunct to insulin in patients with type 1 diabetes: Continuous glucose monitoring data from a 4-week, randomized, placebo-controlled trial (EASE-1). Diabetes Technol. Ther..

[11] U.S. Government Publishing Office (GPO) (1997). Food and Drug Administration Modernization Act of 1997.

[12] Guidance for Industry: Exposure-Response Relationships—Study Design, Data Analysis, and Regulatory Applications. https://www.fda.gov/media/71277/download.

[13] Lee J.Y., Garnett C.E., Gobburu J.V., Bhattaram V.A., Brar S., Earp J.C., Jadhav P.R., Krudys K., Lesko L.J., Li F. (2011). Impact of pharmacometric analyses on new drug approval and labelling decisions: A review of 198 submissions between 2000 and 2008. Clin. Pharmacokinet..

[14] Madabushi R., Wang Y., Zineh I. (2019). A holistic and integrative approach for advancing model-informed drug development. CPT Pharmacomet. Syst. Pharmacol..

[15] Perkins B.A., Soleymanlou N., Rosenstock J., Skyler J.S., Laffel L.M., Liesenfeld K.H., Neubacher D., Riggs M.M., Johnston C.K., Eudy-Byrne R.J. (2020). Low-dose empagliflozin as adjunct-to-insulin therapy in type 1 diabetes: A valid modelling and simulation analysis to confirm efficacy. Diabetes Obes. Metab..

[16] Pieber T.R., Famulla S., Eilbracht J., Cescutti J., Soleymanlou N., Johansen O.E., Woerle H.J., Broedl U.C., Kaspers S. (2015). Empagliflozin as adjunct to insulin in patients with type 1 diabetes: A 4-week, randomized, placebo-controlled trial (EASE-1). Diabetes Obes. Metab..

[17] Elmokadem A., Riggs M.M., Baron K.T. (2019). Quantitative systems pharmacology and physiologically-based pharmacokinetic modeling with mrgsolve: A hands-on tutorial. CPT Pharmacomet. Syst. Pharmacol..

[18] Mondick J., Riggs M., Kaspers S., Soleymanlou N., Marquard J., Nock V. (2018). Population pharmacokinetic- pharmacodynamic analysis to characterize the effect of empagliflozin on renal glucose threshold in patients with type 1 diabetes mellitus. J. Clin. Pharmacol..

[19] Gastonguay M.R. Full covariate models as an alternative to methods relying on statistical significance for inferences about covariate effects: A review of methodology and 42 case studies. Proceedings of the 20th Annual Meeting of the Population Approach Group Europe (PAGE) 2011.

[20] Nguyen T.H.T., Mouksassi M.S., Holford N., Al-Huniti N., Freedman I., Hooker A.C., John J., Karlsson M.O., Mould D.R., Perez Ruixo J.J. (2017). Model evaluation of continuous data pharmacometric models: Metrics and graphics. CPT Pharmacomet. Syst. Pharmacol..

[21] Parke J., Holford N.H., Charles B.G. (1999). A procedure for generating bootstrap samples for the validation of nonlinear mixed-effects population models. Comput. Methods Programs Biomed..

[22] Yafune A., Ishiguro M. (1999). Bootstrap approach for constructing confidence intervals for population pharmacokinetic parameters. I: A use of bootstrap standard error. Stat. Med..

[23] Baron K.T., Macha S., Broedl U.C., Nock V., Retlich S., Riggs M. (2016). Population pharmacokinetics and exposure–response (efficacy and safety/tolerability) of empagliflozin in patients with type 2 diabetes. Diabetes Ther..

[24] Gelman A., Carlin J.B., Stern H.S., Dunson D.B., Vehtari A., Rubin D.B. (2013). Bayesian Data Analysis.

[25] FDA Briefing Document: Endocrinologic and Metabolic Drugs Advisory Committee Meeting. https://www.fda.gov/media/132422/download.

[26] Marshall S.F., Burghaus R., Cosson V., Cheung S.Y., Chenel M., DellaPasqua O., Frey N., Hamren B., Harnisch L., Ivanow F. (2016). Good practices in model-informed drug discovery and development: Practice, application, and documentation. CPT Pharmacomet. Syst. Pharmacol..

