# Supplementary material for: A Model-Informed Drug Development (MIDD) Approach for a Low Dose of Empagliflozin in Patients with Type 1 Diabetes
Source: Pharmaceutics. 2021 Apr 2;13(4):485. doi: 10.3390/pharmaceutics13040485 (PMC8066500; doi:10.3390/pharmaceutics13040485)
Supplement: Supplementary file 1 [file pharmaceutics-13-00485-s001.pdf]

pharmaceutics

MDPI

Supplementary Materials: A Model-Informed Drug Development (MIDD) Approach for a Low Dose of Empagliflozin in Patients with Type 1 Diabetes

Curtis K. Johnston, Rena J. Eudy-Byrne, Ahmed Elmokadem, Valerie Nock, Jan Marquard, Nima Soleymanlou, Matthew M. Riggs and Karl-Heinz Liesenfeld

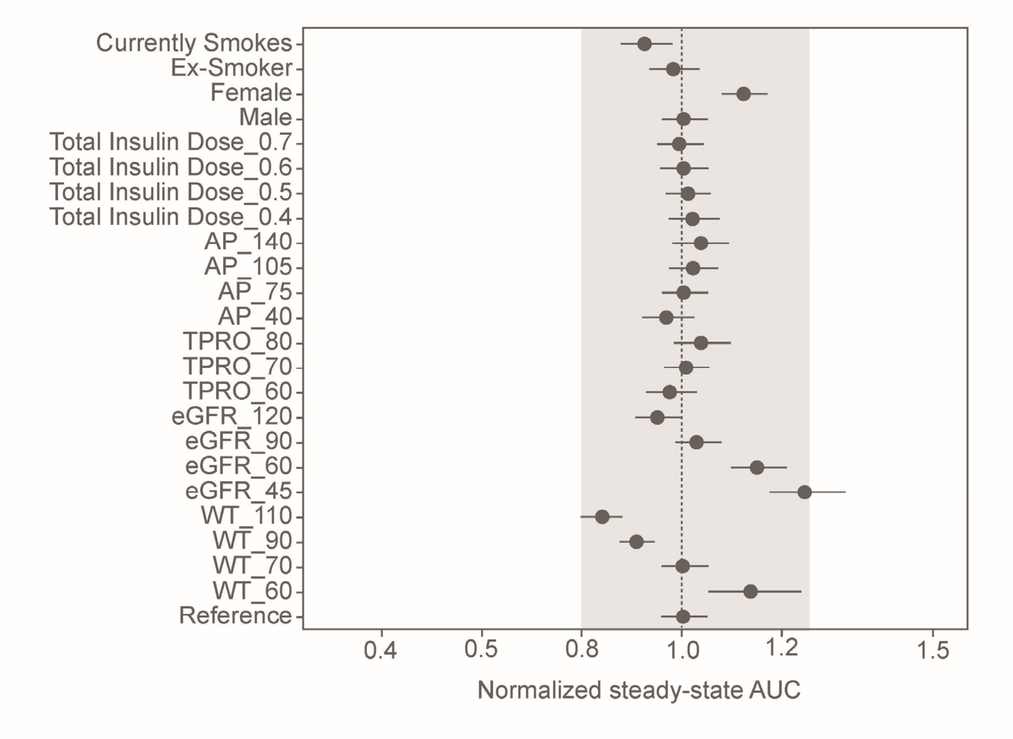

Figure S1. Covariate forest plot on normalized AUC_{ss} for the final PK model. Point ranges represent the median (point) and 95% confidence interval (range) for the covariate effect based upon 500 simulations including parameter uncertainty. The shaded area marks covariate effect from 0.8 to 1.25. Reference subject: male, nonsmoker, total insulin dose = 0.6 IU/kg, AP = 73 IU/kg, TPRO = 68 g/L, eGFR = 99 mL/min/1.73 m^2, and weight = 70 kg. AP, alkaline phosphatase; AUC, area under the curve; AUC_{ss}, area under the curve at steady-state; eGFR, estimated glomerular filtration rate; PK, pharmacokinetic; TPRO, total protein; WT, patient weight.

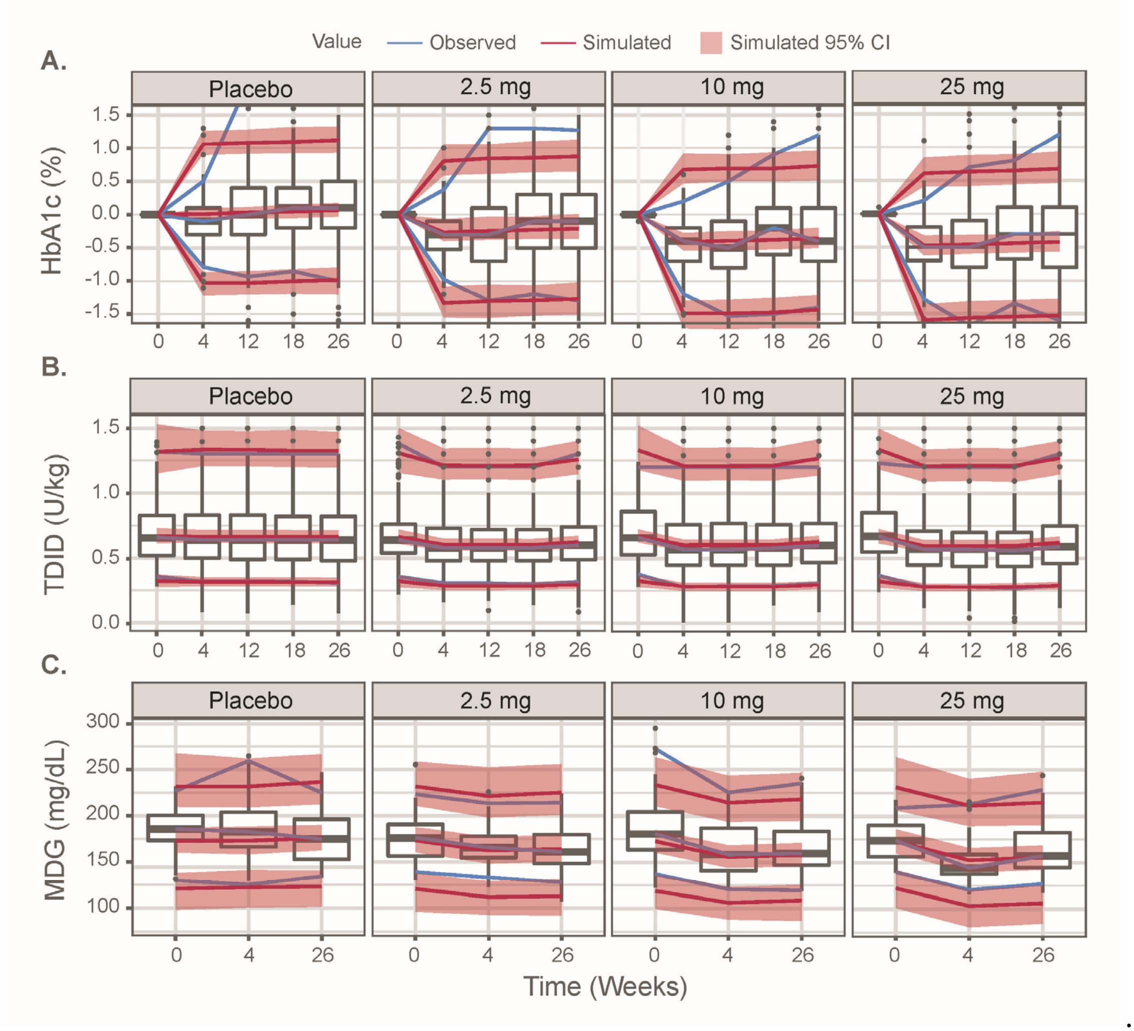

Figure S2. M-EASE-1: External model evaluation for EASE-3 (out-of-sample) by longitudinal visual predictive check by dose for (**a**) HbA1c, (**b**) TDID, and (**c**) MDG. Red lines represent the 96.5th, 50th, and 2.5th percentiles over 500 simulations. The red area is the 95% CI associated with these metrics. The interval between the 97.5th and 2.5th percentile is the 95% prediction interval. Clue lines represent the corresponding observed metrics. Whiskers on box plots represent 1.5× the IQR, with black dots representing observed data falling outside of 1.5× the IQR. CI, confidence interval; HbA1c, glycated hemoglobin; IQR, interquartile range; MDG, mean daily glucose; TDID, total daily insulin dose.

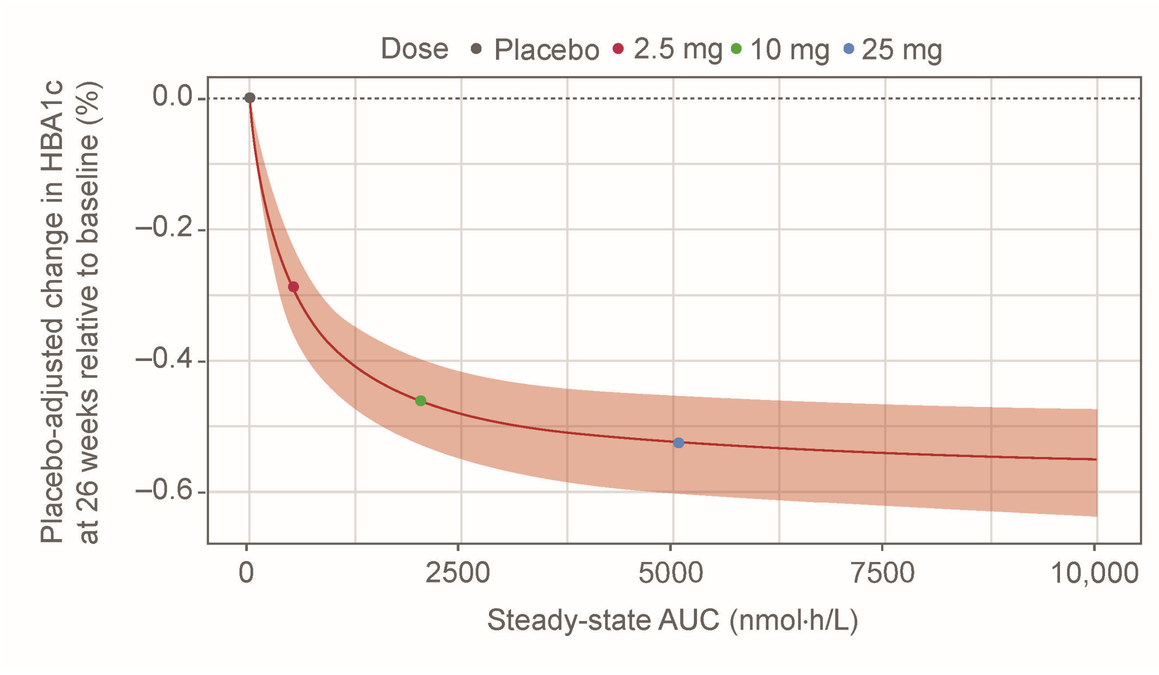

Figure S3. M-EASE-2: Placebo-adjusted simulated change in HbA1c at 26 weeks as a function of empagliflozin AUC_{ss}. Red line and shaded area represent simulated median and associated 95% CI (500 simulations incorporating parameter uncertainty). Colored dots denote the simulated median AUC for each dose. Typical subject: male sex, MDI insulin, eGFR = 98 mL/min/1.73 m^2, baseline weight = 82 kg, baseline total daily dose = 0.660 U/kg, and HbA1c = 8.1%. AUC, area under the curve; AUC_{ss}, area under the curve at steady-state; CI, confidence interval; eGFR, estimated glomerular filtration rate; HbA1c, glycated hemoglobin; MDI, multiple daily injections.

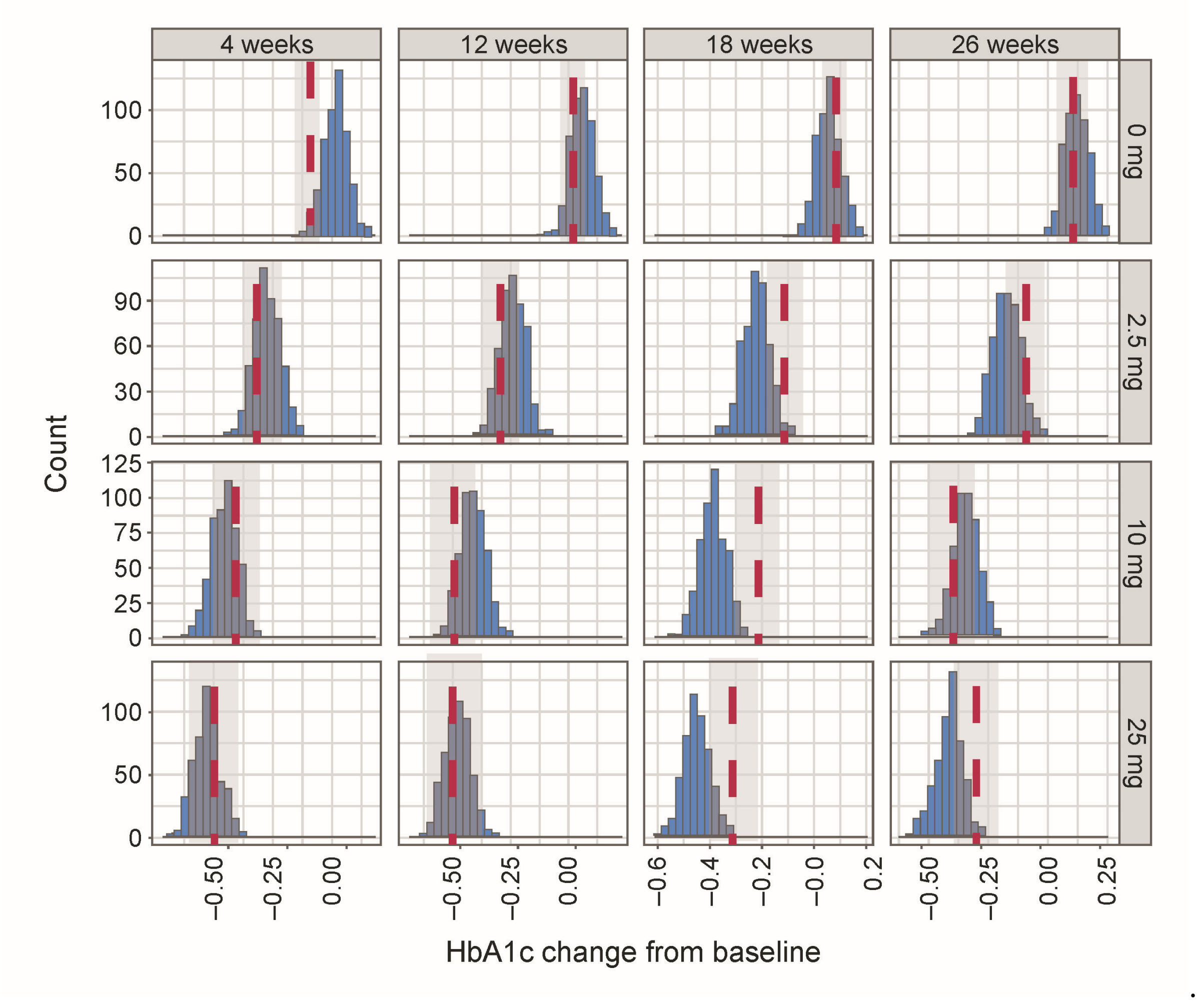

Figure S4. M-EASE-2: Posterior predictive check for EASE-3 (out-of-sample) changes from baseline HbA1c by dose and week**.** Bar graphs are based on 500 simulations. The red line indicates the observed median delta value. The shaded interval indicates ±1.96 SE of the observed data. HbA1c, glycated hemoglobin; SE, standard error.

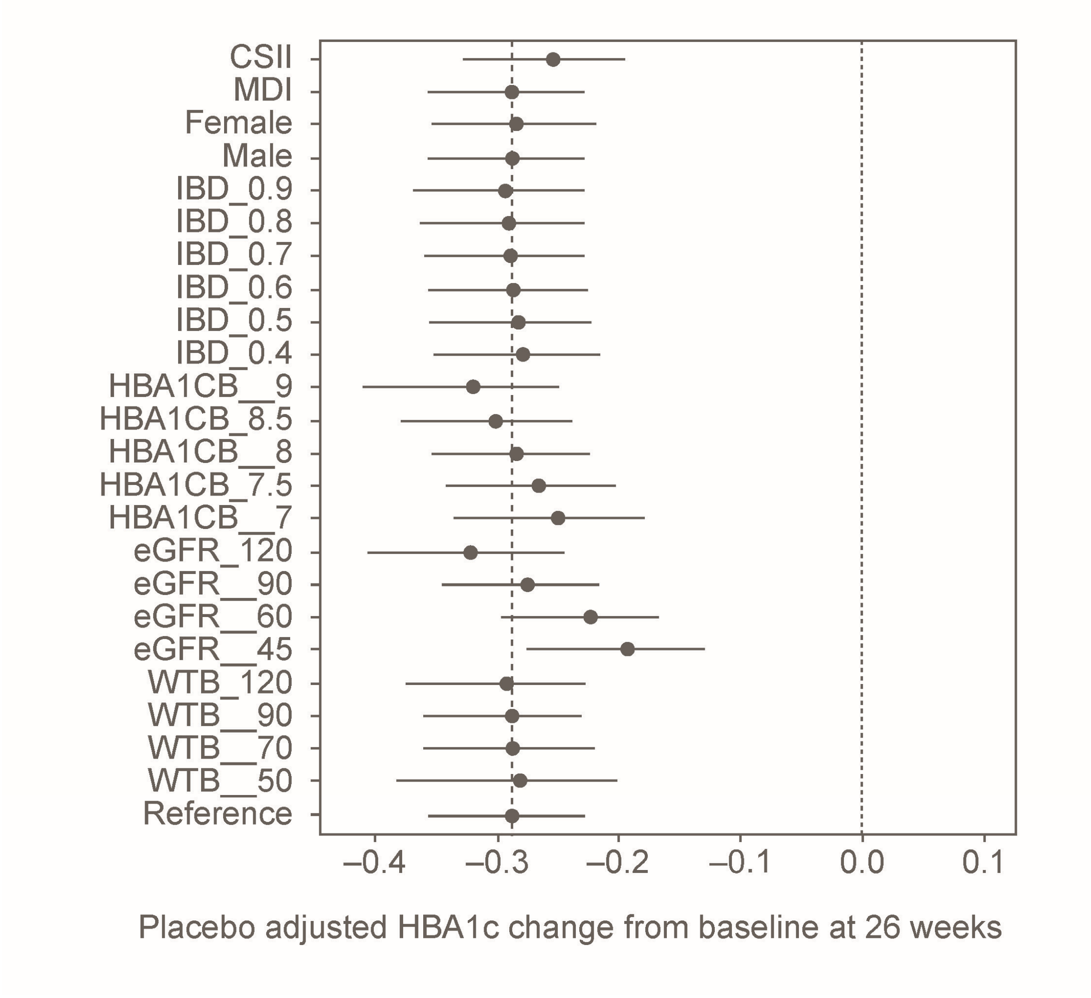

Figure S5. M-EASE-2: Forest plot depicting the relative difference and precision of covariate effects on placebo-adjusted 26-week HbA1c change from baseline. Point ranges represent the median (point) and 95% confidence interval (range from 500 simulations) for the covariate effect. Reference: AUC_{ss} = median of 2.5 mg, male, nonsmoker, MDI insulin, eGFR = 98 mL/min/1.73 m^2, WTB = 82 kg, IDB = 0.660, and HbA1cB = 8.1%. AUC_{ss}, area under the curve at steady-state; CSII, continuous subcutaneous insulin infusion; eGFR, estimated glomerular filtration rate; HbA1c, glycated hemoglobin; HbA1cB, baseline glycated hemoglobin; IDB, total daily insulin dose at baseline; MDI, multiple daily injections; WTB, baseline patient weight.

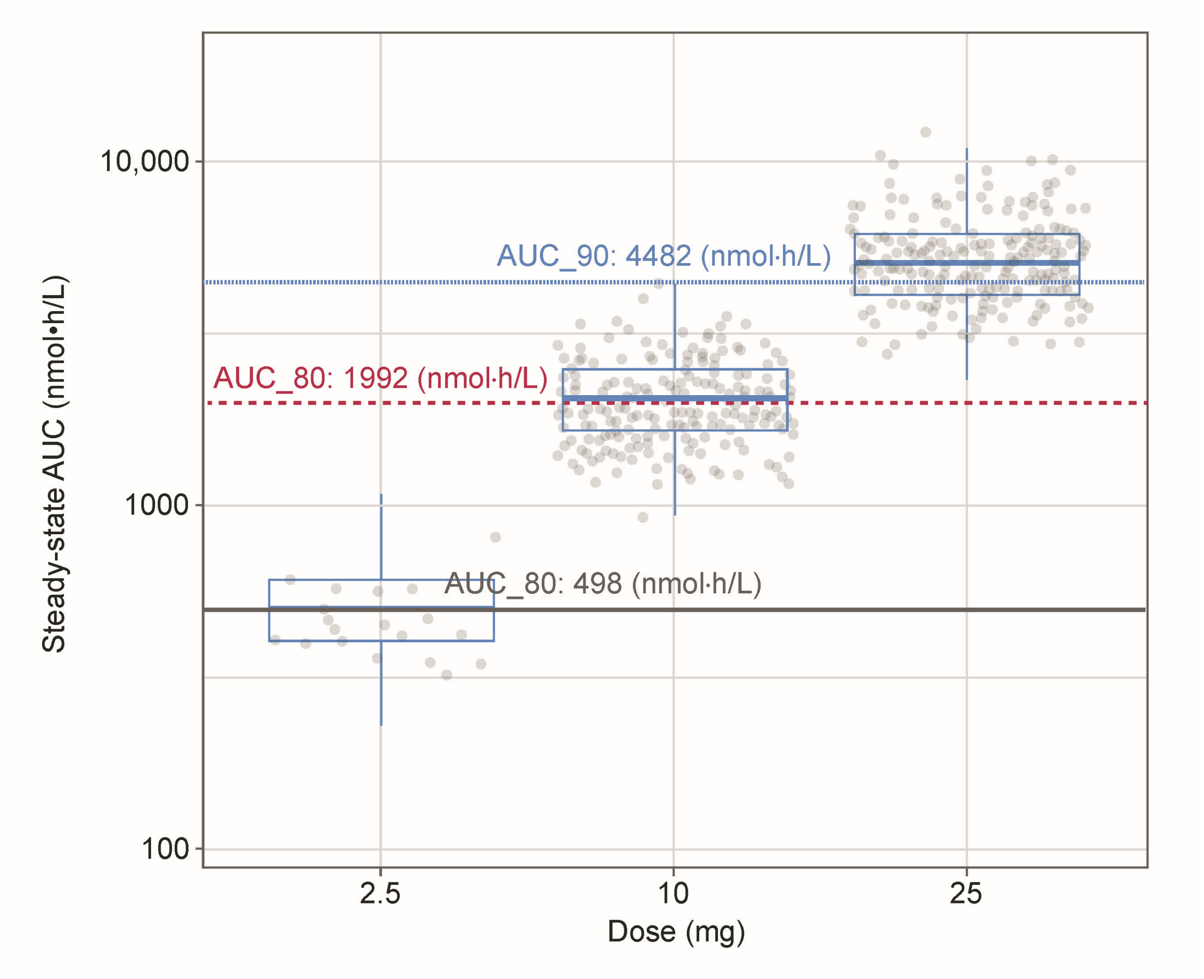

Figure S6. Simulated empagliflozin AUC_{ss} by dose using final population pharmacokinetic model. Gray points represent individual observed steady-state AUC values. Box plots summarize simulated steady-state exposures. AUC, area under the curve; AUC_{ss}, area under the curve at steady-state.

Table S1. Model assumptions.

**M-EASE-2**	
**Assumption**	**E_{max} model was supported by prior information from T2D data for AUC_{50} parameter**
Justification	Overall, estimated pharmacodynamic parameters were comparable between patients with T1D and T2D. Slight differences in G_{max}, I_{max}, and IC_{50} led to an increase in urinary glucose excretion in patients with T1D[17].
Test	Evaluate ability of estimated model to capture the time course of HbA1c via out-of-sample predictions into EASE-3. Sensitivity analyses (varied informativeness and mean) were used to evaluate the impact of the chosen prior for AUC_{50}.

Evaluation	The time course of HbA1c could be sufficiently described, and the sensitivity analyses demonstrated the need for and conservativeness of the chosen prior.
**Assumption**	**A linear placebo effect over the course of treatment was adequate/appropriate**
Justification	A significant decrease in HbA1c was observed during the pretreatment optimization phase. This decrease was not maintained over the course of the study.
Test	Evaluate the ability of the estimated model to capture the time course of HbA1c via out-of-sample predictions into EASE-3 and compare model relative to more complex functional forms.
Evaluation	The time course of the placebo effect could be sufficiently described for internal and external data.
**M-EASE-1**	
**Assumption**	**Change in TDID can be described by empagliflozin drug effect**
Justification	Due to a lack of information regarding the resolution in the time courses of changes in MDG and insulin and meal or exercise information, TDID was estimated independently from MDG. Therefore, the association of insulin reduction and changes in glucose levels was not considered mandatory to describe the impact of empagliflozin on the longer-term insulin dose changes.
Test	Internal and external model evaluation.
Evaluation	TDID data were appropriately described for internal and external data.
**Assumption**	**Change in HbA1c can be described by MDG levels**
Justification	MDG levels are affected by behavioral factors such as food intake and exercise, which are implicitly accounted for in the model.
Test	Internal and external model evaluation.
Evaluation	HbA1c change was appropriately described for internal and external data.

**Assumption**	**A linear placebo effect over the course of treatment was applied**
Justification	Pretreatment optimization in EASE-2 caused a significant decrease in HbA1c that could not be maintained throughout the study. An increase from Week 4 onward was observed in all randomization groups.
Test	Nonlinear placebo models were tested as part of the indirect response model.
Evaluation	The time course of the placebo effect could be sufficiently described for internal and external data.

AUC_{50}, AUC_{ss} at which half the maximal effect; AUC_{ss}, area under the curve at steady-state; E_{max}, maximal effect parameter for empagliflozin AUC_{ss} on TDID and MDG; G_{max}, maximum serum glucose concentration; HbA1c, glycated hemoglobin; IC_{50}, half maximal inhibitory concentration; I_{max}, maximum inhibition; MDG, mean daily glucose; T1D, type 1 diabetes; T2D, type 2 diabetes; TDID, total daily insulin dose.

Table S2. Full covariate PK model: Summary of model parameter estimates.

**Parameter**	**Estimate**	**Unit**	**% RSE**	**95% CI[a]**	**Mapping**
**PK model**					
CL/F	11.2	L/h	2.32	10.8, 11.6	θ_1
V_2/F	1.69	L	24.0	0.105, 5.69	θ_2
Q/F	6.14	L/h	6.10	4.92, 7.30	θ_3
V_3/F	82.2	L	7.55	75.5, 93.2	θ_4
K_a	0.233	1/h	3.36	0.212, 0.259	θ_5
Duration of zero-order input	0.623	h	5.92	0.00209, 0.878	θ_6
ALAG depot	0.135	h	6.32	0.0968, 0.263	θ_7
Sex: CL/F (Female)	0.892		2.71	0.853, 0.935	θ_8
Sex: V_2/F (Female)	0.986		9.56	0.182, 1.68	θ_9

Sex: V_3/F (Female)		0.762		8.88	0.669, 0.874	θ_{10}
Sex: K_a (Female)		1.05		3.73	0.985, 1.12	θ_{11}
Ex-Smoker: (nonsmoker)	CL/F	1.02		1.96	0.986, 1.06	θ_{12}
Cur-Smoker: (nonsmoker)	CL/F	1.08		2.08	1.04, 1.13	θ_{13}
Age: V_2/F		−1.54		10.1	−4.84, −0.412	θ_{14}
Age: V_3/F		0.190		47.8	0.0201, 0.348	θ_{15}
Age: K_a		0.0419		137	−0.0784, 0.126	θ_{16}
WT: CL/F		0.394		15.8	0.280, 0.502	θ_{17}
WT: V_2/F		2.57		10.5	1.06, 4.91	θ_{18}
WT: Q/F		1.11		13.9	0.795, 1.42	θ_{19}
WT: V_3/F		0.414		46.2	0.167, 0.701	θ_{20}
TPRO: CL/F		−0.245		40.8	−0.447, 0.0116	θ_{21}
TPRO: V_2/F		−4.27		11.1	−9.90, −0.0730	θ_{22}
TPRO: V_3/F		−0.381		78.3	−0.952, 0.200	θ_{23}
AP: CL/F		−0.0541		38.5	−0.101, −0.00344	θ_{24}
eGFR: CL/F		0.271		11.3	0.212, 0.329	θ_{25}
TDID: CL/F		0.0469		38.7	0.00213, 0.0935	θ_{26}
ω: CL/F		0.0644	25.8 (%CV)	7.39	0.0499, 0.0810	
Cov: CL/F-Q/F		−0.0614	ρ = -0.784	13.2	−0.0764, −0.0429	

ω: Q/F	0.0952	31.6 (%CV)	28.7	0.0471, 0.131
Cov: CL/F-V_3/F	0.0467	ϱ = 0.422	20.3	0.0141, 0.0818
Cov: Q/F-V_3/F	−0.0806	ϱ = -0.599	22.0	−0.122, −0.0315
ω: V_3/F	0.190	45.7 (%CV)	10.8	0.0800, 0.344
ω: K_a	0.0258	16.2 (%CV)	18.5	0.00985, 0.0428
$^{\delta}$: Proportional EASE-3	0.128	37.0 (%CV)	2.05	0.117, 0.136
$^{\delta}$: Proportional EASE-1	0.0796	28.8 (%CV)	2.53	0.0674, 0.0894

[a]From the nonparametric bootstrap.

Full covariate PK model equations in Table S2

$$\frac{CL}{F_i} = \theta_1 \cdot \theta_8{}^{Sex(female)} \cdot \theta_{12}{}^{ExSmoker} \cdot \theta_{13}{}^{CurrentSmoker} \cdot \left(\frac{WT_i(kg)}{70(kg)}\right)^{\theta_{17}} \cdot \left(\frac{TPRO_i(g/L)}{68(g/L)}\right)^{\theta_{21}} \cdot \left(\frac{AP_i(IU/L)}{73(IU/L)}\right)^{\theta_{24}} \cdot \left(\frac{eGFR_i(mL/min/1.73m^2)}{99(mL/min/1.73m^2)}\right)^{\theta_{25}} \cdot \left(\frac{TDID_i(IU/kg)}{0.6(IU/kg)}\right)^{\theta_{26}} \cdot exp^{\eta_{CL/F}}$$

$$\frac{V_2}{F} = \theta_2 \cdot \theta_9{}^{Sex(female)} \cdot \left(\frac{AGE_i(years)}{44(years)}\right)^{\theta_{14}} \cdot \left(\frac{TPRO_i(g/L)}{68(g/L)}\right)^{\theta_{22}} \cdot \left(\frac{WT_i(kg)}{70(kg)}\right)^{\theta_{18}}$$

$$\frac{V_3}{F} = \theta_4 \cdot \theta_{10}{}^{Sex(female)} \cdot \left(\frac{AGE_i(years)}{44(years)}\right)^{\theta_{15}} \cdot \left(\frac{TPRO_i(g/L)}{68(g/L)}\right)^{\theta_{23}} \cdot \left(\frac{WT_i(kg)}{70(kg)}\right)^{\theta_{20}} \cdot exp^{\eta_{V3/F}}$$

$$\frac{Q}{F} = \theta_3 \cdot \left(\frac{WT_i(kg)}{70(kg)}\right)^{\theta_{19}} \cdot exp^{\eta_{Q/F}}$$

$$D1 = \theta_6$$

$$k_a = \theta_5{}^{Sex(female)} \cdot \left(\frac{AGE_i(years)}{44(years)}\right)^{\theta_{16}} \cdot exp^{\eta_{ka}}$$

$$ALAG = \theta_7$$

Age, patient age; ALAG, oral absorption lag time; AP, alkaline phosphatase; CI, confidence interval; CL/F, apparent clearance after oral dosing; Cov, covariate; Cur, current; CV, coefficient of variation; eGFR, estimated glomerular filtration rate; Ka, absorption rate constant; PK, pharmacokinetic; Q/F, apparent (oral) intercompartmental clearance; Sex, patient gender; TDID, total daily insulin dose; TPRO, total protein; V2/F, apparent central volume of distribution after oral dosing; V3/F, apparent peripheral volume of distribution after oral dosing; WT, patient weight; $^{\delta}$, residual variability; RSE, relative standard error; ω, inter-individual variance.

Table S3. M-EASE-1: Summary of final HbA1c/MDG/TDID model parameter estimates.

Parameter	Estimate (%RSE)	95% CI	Units	Mapping
Baseline HbA1c	8.15 (0.375)	8.09, 8.21	%	θ_1
Sex_{HbA1c}	0.99 (0.545)	0.98, 1		θ_2
WT_{HbA1c}	−0.0258 (53.5)	−0.0528, 0.00125		θ_3
$\gamma_{MDG\ EFF}$	0.487 (4.58)	0.445, 0.532		θ_4
$\omega_{Baseline\ HbA1c}$	0.00437 (6.49)	0.00381, 0.00492	6.62 (CV%)	
$Cov_{Baseline\ HbA1c–MDG\ EFF}$	0.0106 (36.3)	0.00306, 0.0182	ϱ = 0.194	
$\omega_{MDG\ EFF}$	0.461 (24.3)	0.242, 0.681	76.5 (CV%)	
$^{\delta}:_{propHbA1c}$	0.00218 (3.76)	0.00202, 0.00234	4.67 (CV%)	
$TDID_{t0}$	0.657 (3.22)	0.617, 0.7	IU/kg	θ_1
WT_{TDID}	0.317 (21.4)	0.184, 0.451		θ_2
Sex_{TDID}	0.96 (2.55)	0.913, 1.01		θ_3
$eGFR_{TDID}$	0.145 (41.8)	0.0261, 0.263		θ_4
$HbA1c_{TDID}$	0.368 (40.5)	0.0759, 0.661		θ_5
INC (EASE-1 only)	1.05 (1.8)	1.01, 1.09		θ_6
WT_{INC}	0.0645 (159)	−0.137, 0.266		θ_7
Sex_{INC}	1.08 (3.38)	1.01, 1.15		θ_8
$eGFR_{INC}$	0.0124 (590)	−0.131, 0.156		θ_9
$HbA1c_{INC}$	−0.546 (30.9)	−0.877, −0.215		θ_{10}

TDID -- E_{max}	0.186 (12.6)	0.145, 0.238		$θ_{11}$
TDID -- AUC_{50}[a]	110 nmol•h/L (104)	14.3, 836		$θ_{12}$
$TDID_{t0EASE2}$[b]	1.02 (3.44)	0.953, 1.09		
$TDIDE_{MAX\_EASE2}$[b]	0.556 (13.8)	0.424, 0.729		
$MDG_{t0–24}$	4.16e+03 mg•day/dL (0.611)	4.11e+03, 4.21e+03		$θ_1$
INS_MDG effect	−0.261 (105)	−0.797, 0.275		$θ_3$
PBO_{MDG}[c]	0.0136 (mg/dL)•24 (47)	0.00544, 0.0343		$θ_3$
$AUC_{50,MDG}$[c]	370 nmol•h/L (75.7)	83.9, 1.63e+03		$θ_4$
$E_{max, MDG}$	634 mg•day/dL (8.74)	534, 753		$θ_5$
WT_{EMAX}	−0.113 (201)	−0.56, 0.333		$θ_6$
SEX_{EMAX}	1.09 (7.14)	0.951, 1.26		$θ_7$
$eGFR_{EMAX}$	0.0707 (128)	−0.107, 0.249		$θ_8$
$INSTD_{EMAX}$	0.995 (7.16)	0.865, 1.14		$θ_9$
ωTDIDBASE	0.0974 (6.48)	0.085, 0.11	32.0 (CV%)	
ωTDIDBASE - $TDIDE_{MAX}$	0.00579 (332)	−0.0319, 0.0435	$\dot{\rho}$ = 0.0215	
$ωTDIDE_{MAX}$	0.554 (16.7)	0.373, 0.736	86.0 (CV%)	
ωINC	0.00858 (30.3)	0.00348, 0.0137	9.28 (CV%)	
$ωMDG_{t0}$	0.009 (10.9)	0.00708, 0.0109	9.51 (CV%)	
ωMDG E_{max}	0.0744 (50.1)	0.0013, 0.148	27.8 (CV%)	

δ: Proportional – TDID	0.0239 (7.19)	0.0205, 0.0273	15.6 (CV%)
δ: Additive – TDID	0.001 (49)	4.03e-05, 0.00196	0.0316 (SD)
δ: Proportional – MDG	0.0254 (4.72)	0.0231, 0.0278	16.0 (CV%)
δ: Additive – MDG	0.001 (16.3)	0.00068, 0.00132	0.0316 (SD)

[a]Estimated from placebo only data and fixed in the estimation of the impact of EMPA on TDID time course. [b]As EASE-2 included a pre-treatment insulin intensification phase and EASE-1 did not, study-specific effects were implemented on baseline insulin dose and the E_{max} parameter to allow for differences seen in observed data due to study design (see equations below). Although the data for the EASE-2 pre-treatment phase were not included in the analysis, the separate parameter effects were considered necessary for this study to account for the different relative starting point for these patients as affected by the pre-treatment difference. [c]Estimated from placebo only data and fixed in the estimation of the impact of EMPA on MDG time course.

Summary of final HbA1c parameters in Table S3 (M-EASE 1)

$$HbA1c_{t0,i} = exp^{\theta_1} \cdot {\theta_3}^{SEX(Female)} \cdot \left(\frac{W\,T_i(kg)}{82(kg)}\right)^{\theta_2} \cdot exp^{\eta_1}$$

$$HbA1c_{i,j} = HbA1c_{t0,i} \cdot \left(\frac{MDG_{i,J}}{MDG_{t0,i}}\right)^{\theta_4 \cdot exp^{\eta_2}}$$

Summary of final TDID parameters in Table S3 (M-EASE 1)

$$TDID_{t,0i} = exp^{\theta_1} \cdot {\theta_3}^{SEX(Female)} \cdot \left(\frac{W\,T_i(kg)}{82(kg)}\right)^{\theta_2} \cdot \left(\frac{eGFR(mL/min/1.73m^2}{99(mL/min/1.73m^2)}\right)^{\theta_4} \left(\frac{Base.HbA1c_i(\%)}{8.1(\%)}\right)^{\theta_5} \cdot TDIDEBASE_{(EASE2)} \cdot exp^{\eta_1}$$

$$inc_i = exp^{\theta_6} \cdot {\theta_8}^{SEX(Female)} \cdot \left(\frac{W\,T_i(kg)}{82(kg)}\right)^{\theta_7} \cdot \left(\frac{eGFR(mL/min/1.73m^2}{99(mL/min/1.73m^2)}\right)^{\theta_9} \left(\frac{Base.HbA1c_i(\%)}{8.1(\%)}\right)^{\theta_{10}} \cdot TDIDEMAX_{(EASE2)} \cdot exp^{\eta_3}$$

$$Emax_{TDID,i} = exp^{(\theta_{11} + TDIDEMAX_{(EASE2)} + \eta_2)} / exp^{(1 + \theta_{11} + TDIDEMAX_{(EASE2)} + \eta_2)}$$

$$AUC_{50,TDID} = \theta_{12}$$

$$TDID_{t,i} = TDID_{t0,i} \cdot Inc_i \cdot \left(1 - \frac{E_{max,TDID,i} \cdot AUC_{ss.i}}{AUC_{50,TDID} + AUC_{ss,i}}\right)$$

Summary of final MDG parameters in Table S3 (M-EASE 1)

$$MDG_{t0,i} = \theta_1 \cdot exp^{\eta_1}$$

$$EMAX_{MDG,i} = exp^{\theta_5} \cdot {\theta_7}^{SEX(Female)} \cdot \left(\frac{W\,T_i(kg)}{82(kg)}\right)^{\theta_6} \cdot \left(\frac{eGFR(mL/min/1.73m^2}{99(mL/min/1.73m^2)}\right)^{\theta_8} \cdot {\theta_9}^{INSDT[CSII]} \cdot exp^{\eta_2}$$

$$AUC_{50,MDG} = \theta_4$$

$$PBO_{MDG} = \theta_3$$

$$MDG_{t,i} = MDG_{t0,i} \cdot \left(\frac{TDID_{t,i}}{TDID_{t0,i}}\right)^{\theta_2} + PBO_{MDG} \cdot TIME - \left(\frac{E_{max,MDG,i} \cdot AUC_{ss,i}}{AUC_{50,MDG} + AUC_{ss,i}}\right)$$

AUC_{50}, AUC_{ss} leading to 50% of maximal effect; AUC_{ss}, area under the curve at steady-state; Base, baseline; CI, confidence interval; Cov, covariance; CSII, continuous subcutaneous insulin infusion; CV, coefficient of variance; EFF, power coefficient; eGFR, estimated glomerular filtration rate; E_{max}, maximal effect parameter for EMPA AUC_{ss} on HbA1c; EMPA, empagliflozin; HbA1c, glycated hemoglobin; INC, scale parameter reflecting the amplitude for insulin dose adjustment (applies only to EASE-1 during treatment week 1); INS, insulin; INSDT, insulin dose type (MDI vs. CSII); MDG, mean daily glucose; MDI, multiple daily injections; PBO, time-dependent MDG placebo effect; RSE, relative standard error; SEX, patient gender; SD, standard deviation; TDID, total daily insulin dose; WT, patient weight; $^{\delta}$, residual variance; γ, insulin effect; ω, inter-individual variance.

Table S4. M-EASE-2: Full covariate model, summary of parameter estimates.

Parameter	Estimate	95% CI	*n* (effective)	Rhat	Mapping
Baseline HbA1c	8.14%	8.07, 8.22	4332	1.001	θ_1
AUC_{50}	498 nmol•h/L	296, 819	25,078	1.000	θ_2
E_{max}	0.579%	0.491, 0.678	6603	1.001	θ_3
Placebo effect	2.61×10^{-5}%/h	1.96×10^{-5}, 3.29×10^{-5}	40,000	1.000	θ_4
Sex – $baseline_{HbA1c}$ (female)	0.988	0.977, 1.00	4707	1.001	θ_5
Sex – E_{max} (female)	0.984	0.827, 1.17	13,259	1.000	θ_6
Sex – placebo (female)	0.727	0.534, 0.971	40,000	1.000	θ_7
INSDT – $baseline_{HbA1c}$ (CSII)	1.00	0.988, 1.01	4754	1.001	θ_8
INSDT – E_{max} (CSII)	0.880	0.737, 1.04	13,152	1.000	θ_9
INSDT – placebo (CSII)	1.47	1.10, 1.99	40,000	1.000	θ_{10}
WTB – $baseline_{HbA1c}$	−0.0311	−0.0612, -0.00102	4680	1.001	θ_{11}
WTB – E_{max}	0.0555	−0.351, 0.458	13,343	1.000	θ_{12}
eGFR – $baseline_{HbA1c}$	0.0123	−0.0157, 0.0403	4842	1.002	θ_{13}
eGFR – E_{max}	0.504	0.116, 0.917	16,235	1.000	θ_{14}
IDB – $baseline_{HbA1c}$	0.0141	−0.00425, 0.0326	4874	1.001	θ_{15}
IDB – E_{max}	0.0552	−0.190, 0.300	13,939	1.000	θ_{16}
$Baseline_{HbA1c}$ – E_{max}	0.999	−0.358. 2.33	2983	1.001	θ_{17}
$^{\delta}$: Proportional	0.00210	0.00196, 0.00222	40,000	1.000	
$^{\delta}$: Additive	0.0112	0.00705, 0.0175	40,000	1.000	
ω: $Baseline_{HbA1c}$	0.00515	0.00459, 0.00579	40,000	1.000	
Cov: $Baseline_{HbA1c}$ – E_{max}	−0.00159	−0.00643, 0.00414	2403	1.002	
ω: E_{max}	0.137	0.0767, 0.221	831	1.005	

Reference: male, MDI, eGFR = 98 mL/min/1.73 m^2, patient weight = 82 kg, total daily insulin dose = 0.66 U/kg, and HbA1c = 8.1%.

Equations (Supplementary Table S4)

$$Baseline_{HbA1c} = exp^{\theta_1} \cdot \theta_5{}^{SEX(Female)} \cdot \theta_8{}^{INSDT[CSII]} \cdot \left(\frac{W\,T_i(kg)}{82(kg)}\right)^{\theta_{11}} \cdot \left(\frac{eGFR(mL/min/1.73m^2}{98(mL/min/1.73m^2)}\right)^{\theta_{13}} \cdot \left(\frac{IDB_i(IU/kg)}{0.660(IU/kg)}\right)^{\theta_{15}} \cdot exp^{\eta_1}$$

$$AUC_{50} = exp^{\theta_2}$$

$$EMAX_i = exp^{\theta_3} \cdot \theta_6{}^{SEX(Female)} \cdot \theta_9{}^{INSDT[CSII]} \cdot \left(\frac{W\,T_i(kg)}{82(kg)}\right)^{\theta_{12}} \cdot \left(\frac{eGFR(mL/min/1.73m^2}{98(mL/min/1.73m^2)}\right)^{\theta_{14}} \cdot \left(\frac{IDB_i \cdot (IU/kg)}{0.660(IU/kg)}\right)^{\theta_{16}} \cdot \left(\frac{Base.HbA1c_i(\%)}{8.1(\%)}\right)^{\theta_{17}} \cdot exp^{\eta_2}$$

$$Placebo = exp^{\theta_4} \cdot \theta_7{}^{Sex[female]} \cdot \theta_{10}{}^{INSDT[CSII]} \cdot TIME$$

AUC_{50}, AUC_{ss} leading to 50% of maximal effect; AUC_{ss}, area under the curve at steady-state; CI, confidence interval; Cov, covariance; CSII, continuous subcutaneous insulin infusion; eGFR, estimated glomerular filtration rate; E_{max}, maximal effect parameter for empagliflozin AUC_{ss} on HbA1c; HbA1c, glycated hemoglobin; IDB, total daily insulin dose at baseline; INSDT, insulin dose type (multiple daily injections vs CSII); Sex, patient gender; WTB, baseline patient weight; $^{\delta}$, residual variability; ω, inter-individual variability.

Table S5. M−EASE−2.

A) Impact of prior variance on placebo−adjusted predicted median HbA1c change from baseline at 26 weeks.

Model	Median	95% CI
Final model	−0.285	−0.386, −0.188
10x variance (AUC_{50})	−0.361	−0.488, −0.235
50x variance (AUC_{50})	−0.411	−0.532, −0.260
100x variance (AUC_{50})	−0.421	−0.548, −0.266
Noninformative variance (AUC_{50})	−0.467	−0.566, −0.321
Fixed (AUC_{50})	−0.254	−0.347, −0.162

B) Impact of prior mean on placebo−adjusted predicted median HbA1c change from baseline at 26 weeks.

Model	Median	95% CI
Final model	−0.285	−0.386, −0.188
Extreme large mean (AUC_{50})	−0.126	−0.225, −0.0309
Extreme small mean (AUC_{50})	−0.484	−0.580, −0.391
50% increased mean (AUC_{50})	−0.262	−0.372, −0.153
50% decrease mean (AUC_{50})	−0.334	−0.450, −0.232

Extreme large mean: 22,026 nmol•h/L; Extreme small mean: 0.00005 nmol•h/L.

C) M−EASE−2: Impact of prior variance on estimated AUC_{50} (nmol•h/L).

Model	Median	95% CI
Final model	498	296, 819
10x variance (AUC_{50})	237	62.3, 610
50x variance (AUC_{50})	114	5.21, 476
100x variance (AUC_{50})	72.0	0.655, 447
Noninformative variance (AUC_{50})	1.30	1.50e−07, 286

D) M−EASE−2: Impact of prior mean on estimated AUC_{50} (nmol•h/L).

Model	Median	95% CI
Final model	498	(296, 819)

Extreme large mean (AUC_{50})	3.47e+03	(2.12e+03, 6.19e+03)
Extreme small mean (AUC_{50})	4.55e−05	(2.44e−05, 8.43e−05)
50% increased mean (AUC_{50})	648	(393, 1.03e+03)
50% decrease mean (AUC_{50})	305	(173, 517)

Extreme large mean: 22,026 nmol•h/L; Extreme small mean: 0.00005 nmol•h/L. AUC_{50}, area under the concentration–time curve at steady−state leading to 50% of maximal effect; CI, confidence interval; HbA1c, glycated hemoglobin.
